# Cost-effectiveness of national health insurance programs in high-income countries: A systematic review

**DOI:** 10.1371/journal.pone.0189173

**Published:** 2017-12-15

**Authors:** Son Nghiem, Nicholas Graves, Adrian Barnett, Catherine Haden

**Affiliations:** 1 Institute of Health and Biomedical Innovation Queensland University of Technology, Brisbane, Queensland, Australia; 2 Library Queensland University of Technology, Brisbane, Queensland, Australia; Public Library of Science, FRANCE

## Abstract

**Objectives:**

National health insurance is now common in most developed countries. This study reviews the evidence and synthesizes the cost-effectiveness information for national health insurance or disability insurance programs across high-income countries.

**Data sources:**

A literature search using health, economics and systematic review electronic databases (PubMed, Embase, Medline, Econlit, RepEc, Cochrane library and Campbell library), was conducted from April to October 2015.

**Study selection:**

Two reviewers independently selected relevant studies by applying screening criteria to the title and keywords fields, followed by a detailed examination of abstracts.

**Data extraction:**

Studies were selected for data extraction using a quality assessment form consisting of five questions. Only studies with positive answers to all five screening questions were selected for data extraction. Data were entered into a data extraction form by one reviewer and verified by another.

**Evidence synthesis:**

Data on costs and quality of life in control and treatment groups were used to draw distributions for synthesis. We chose the log-normal distribution for both cost and quality-of-life data to reflect non-negative value and high skew. The results were synthesized using a Monte Carlo simulation, with 10,000 repetitions, to estimate the overall cost-effectiveness of national health insurance programs.

**Results:**

Four studies from the United States that examined the cost-effectiveness of national health insurance were included in the review. One study examined the effects of medical expenditure, and the remaining studies examined the cost-effectiveness of health insurance reforms. The incremental cost-effectiveness ratio (ICER) ranged from US$23,000 to US$64,000 per QALY. The combined results showed that national health insurance is associated with an average incremental cost-effectiveness ratio of US$51,300 per quality-adjusted life year (QALY). Based on the standard threshold for cost-effectiveness, national insurance programs are cost-effective interventions.

**Conclusions:**

Although national health insurance programs have been introduced in most developed countries, only a few studies have examined their cost-effectiveness. All the selected studies revealed strong evidence to support health insurance programs or health reforms in the United States. The average ICER in this study is below the standard threshold for cost-effectiveness used in the US. The small number of relevant studies is the main limitation of this study.

## Introduction

National health insurance programs are motivated by the principles of pooled risk and economies of scale. In particular, substantial resources mobilized from the whole population, or a large part of the population, enable health services to be delivered at reasonable costs. Opponents of national health insurance schemes argue that the private market is the best tool to deliver health services at efficient prices [1]. However, due to rapid inflation and moral hazard behaviors of providers, such as exploiting patients by wielding market power and advising them to consume more services than necessary, market prices render health care unaffordable to a large part of the population [2].

Provision of efficient health services for nationally-insured patients is a desirable policy goal, but evidence about the performance of these schemes is mainly anecdotal and subjective [3]. Examining the cost-effectiveness of national health insurance programs is a key way to address this issue.

National health insurance programs have been implemented in most developed, and even some developing countries [4]. The growth of national health insurance programs suggests that they have been effective in improving health services or lowering costs. For example, a comparison between healthcare administrative costs in the United States and Canada revealed that the Canadian universal healthcare system played a role in lowering costs [5]. Such specific studies are useful, but there has been no systematic review of the cost-effectiveness of national health insurance programs in higher-income countries, hence the need for this study.

Our objective was to determine systematically the cost-effectiveness of providing national health insurance from large-scale studies in high-income countries, which we define as member countries of the Organization for Economic Co-operation and Development (OECD). This study hypothesizes that national health insurance programs are cost-effective. We also aimed to synthesize results of previous studies to generalize the overall effectiveness of national health insurance.

## Methods

### Protocol

This study was registered in April 2015 at PROSPERO, the International Prospective Register of Systematic Reviews, based at the Centre for Reviews and Dissemination (CRD) at the University of York; (ID: CRD42015019897). The protocol can be accessed from the URL:http://www.crd.york.ac.uk/PROSPERO/display_record.asp?ID=CRD42015019897. As part of the review, it was unnecessary to convert monetary measure to purchasing power parity because all relevant studies found were located in the US.

### Scope of the study

We focused on searching for studies that examined the overall cost-effectiveness of national health insurance programs. The search was limited to English-language publications. We included both peer-reviewed articles and gray literature, including working papers and conference papers. The search scope focused on health and economic literature databases. We extended the search to include databases of systematic reviews, and those indexing pharmaceutical and insurance literature, as well as manually-searching the references of relevant articles.

### Search strategy

Searches were conducted from April to October 2015 in the popular medical literature databases PubMed and Embase/Medline (via embase.com). We also searched the economic databases Econlit (via Proquest) and RepEc, the citation databases Scopus and Web of Science, and the databases of systematic reviews:Cochrane Library, DARE, NHS EDD and HTA (in the CRD at the University of York), Campbell Library (Campbell Collaboration), EPPI Centre Database of Promoting Health Effectiveness Reviews (DoPHER) and the Health Economics Evaluation Database (HEED).

The main search phrase comes from the title of this review: *cost-effectiveness of national health insurance programs*. Synonyms of keywords in the search phrase were used to increase the search results. We used Boolean operators and wildcards to combine keywords and their synonyms in a general search phrase. The following keyword search was run using the Boolean operators OR and AND, and using phrase searching and truncation: "cost benefit*" OR "cost effectiveness" OR economic*) *AND* (*"disability insurance" OR "national health" OR "health insurance"*.

This approach ensured that papers using the synonyms *global health insurance* and *national disability insurance* for *national health insurance* would be captured. The cost-effectiveness element of the search was covered by using the keyword economic*, ensuring that papers covering either *economic analysis* or *economic evaluation* would be found, and *cost benefit* was used as an alternative term for *cost effectiveness*. We performed initial searches within the title and abstract fields.

The original search found nearly 60,000 articles but only 22,000 remained after removing duplications (S1 Table). The use of wildcards in the search phrase contributed to a large number of returns. Thus, we filtered the search results by using exact matches for keywords *health insurance* and *cost effectiveness* in the title and abstract, resulting in just 18 hits. We scanned the reference lists and contacted authors of relevant articles, but no additional articles were found. For evidence synthesis, we selected studies that included an ICER and found only four eligible studies (see the Transparent Reporting of Systematic Review and Meta-Analysis (PRISMA) diagram in S1 Fig).

### Study selection

Many studies examined alternative outcomes (e.g., effects of national health insurance on employment or life expectancy), and thus were excluded. We also excluded many descriptive and methodological studies, as well as textbooks and guidelines. Studies of micro health insurance programs in developing countries were not selected because their results are not comparable with national health insurance schemes in developed nations. For example, a cost-effectiveness study of community-based health insurance programs in Burkina Faso [6] estimated an ICER of only US$1,000. Randomized control trials and those focused on specific diseases were also excluded due to the difficulty of generalizing the results to national populations.We are aware that results from randomized controlled trials are classified as the most reliable by the Oxford 2011 Levels of Evidence [7] but the scale of this project is beyond that covered in most clinical trials.

After applying these criteria, only the 18 most relevant articles (see S3 Table) were examined in detail to extract data for synthesis.

### Quality assessment

In order to assess the quality of the studies, we modified the Grading of Recommendations Assessment, Development and Evaluation (GRADE) [8] recommendations, using criteria that had five yes/no questions (see Table 1). This was applied to the 18 search results, and studies that met all selection criteria were included in the evidence synthesis. We excluded 14 studies that failed to meet Criterion 5, ‘produce an incremental cost-effectiveness ratio (ICER)’, and one study that did not use QALY as an output. Thus, the evidence synthesis was conducted using the four studies that remained [9–12]. The small number of relevant studies found in this review is in line with a recent Cochrane review [13], which found only two relevant studies in the US on strategies to expand health insurance programs to the vulnerable population.

**Table 1:** Quality assessment tool: studies were included only if the answer to all five questions was ‘yes’.

No.	Questions
1	Was the study’s purpose clearly explained as cost-effectiveness analysis?
2	Were the target population and comparators described?
3	Did the study conduct an analysis of uncertainty?
4	Were costs of the interventions measured?
5	Did the study produce an incremental cost-effectiveness ratio?

**Fig 1:**
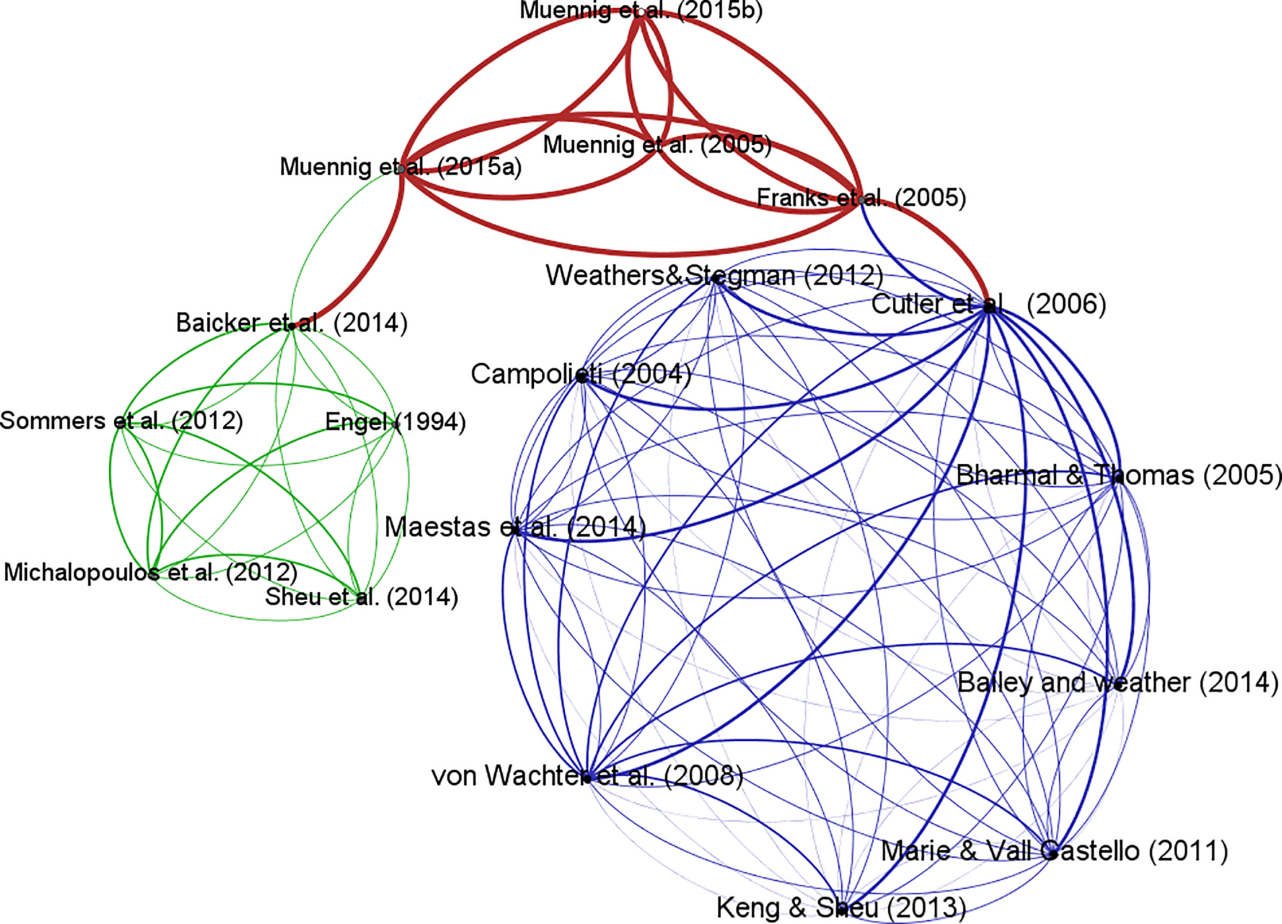
Network of articles, the thickness of the lines represents the weight of the study calculated from answers to the five questions in Table 1.

The connections between the 18 studies are shown in Fig 1 where linkages are represented by methodology, and the thickness of the lines is the weight of studies based on the five questions in Table 1, with each ‘yes’ answer being given a score of 1. It can be seen in Fig 1 that all four Cost-Effectiveness Analysis (CEA) studies in the middle group achieved the maximum score. Among studies with a less than perfect score, the right group includes studies that use econometrics analyses;while those in the left group use other methods (e.g., Randomized Control Trials and natural experiments). The linkages between the CEA group and other groups were plotted using topics: *the Oregon health experiment* (Baicker et al., 2014) and *Medicare* (Cutler et al., 2006).

Data from the four selected studies were extracted for result synthesis. The data extraction form included authors and year of publication, methods used, countries, scale, outputs, interventions and outcomes (see Table 2 below and the S2 Table for a summary of the selected studies). Although we are mainly interested in the outcomes of a cost-effectiveness analysis, other variables were collected to identify factors associated with their effectiveness. Two variables, costs and outcomes, were extracted separately for the control and treatment groups so that we could draw their distributions to synthesize results of the ICER.

**Table 2:** Study details recorded by the data extraction form.

Variables	Definitions
Author/year	Authors of the study and year of publication
Methods	Cost-effectiveness analysis, Econometric analysis, others
Types of publications	Articles, reports, working papers, others
Country	Country that the study investigated
Base period	Period (year) in which prices are used as a reference
Scale	Country, states, regions, others
Study design	RTC, before-after, with-without, double difference, others
Participants	Whole population, target groups, others
Interventions	Health insurance, disability insurance, social insurance, others
Costs of control	Costs associated with health care or disability care
Costs of treatment	Costs associated with health care or disability care
Outcomes of control	Quality-adjusted life years
Outcomes of treatment	Quality-adjusted life years
ICER	Incremental cost-effectiveness ratio

Possible biases when synthesizing results across studies include effects of inflation and exchange rate to monetary values. Fortunately, all the selected studies are from the US, so the effect of exchange rates is nil. Two selected studies ([9, 11]) used 2013 prices and two studies ([12, 10]) used 1994 prices.Thus, we converted the ICER of these two studies to 2013 prices using the US Consumer Price Index (CPI) [14] (the results were similar when using the Personal Consumption Expenditure Health index).

### Evidence synthesis

To estimate an overall picture of cost-effectiveness, results of selected studies were synthesized using a Monte Carlo simulation. The costs and QALYs of the control and treatment groups were randomly sampled from distributions, with the aim of covering the likely range of costs and QALYs based on the available evidence. We used log-normal distributions for costs and QALYs, employing the means and standard deviations given in the articles. The choice of a log-normal distribution aimed to reflect the common features of non-negative and positive skew of costs and QALYs [15]. The log-normal distribution has been used in the synthesis of previous systematic reviews on the cost-effectiveness of medical intervention [16].

The evidence synthesis of cost-effectiveness was conducted using costs and effects of simulated data for the control (no health insurance) and treatment (with health insurance) groups. For example, simulated data are used to estimate the mean and confidence interval of the ICER. The probability of national health insurance being cost-effective, against willingness-to-pay was also plotted.

## Results

The four selected studies all revealed that national health insurance programs and health reforms in the USA are cost-effective based on the standard threshold of willingness-to-pay for a QALY of $50,000–$100,000 [17–19]. The ICER ranged from $24,000 for the expansion of Medicare coverage [12], to $62,000 for healthcare reforms [11]. One study revealed that health insurance produced an ICER of $35,000 [10]. The Oregon Health Study, which randomly provided Medicaid for uninsured individuals, had an estimated ICER of $62,000. One [11] of the four studies examined the effects of welfare reforms rather than health insurance. However, we included this study in the analysis as it affects a vulnerable group that could benefit from an expanding national health insurance.

**Fig 2:**
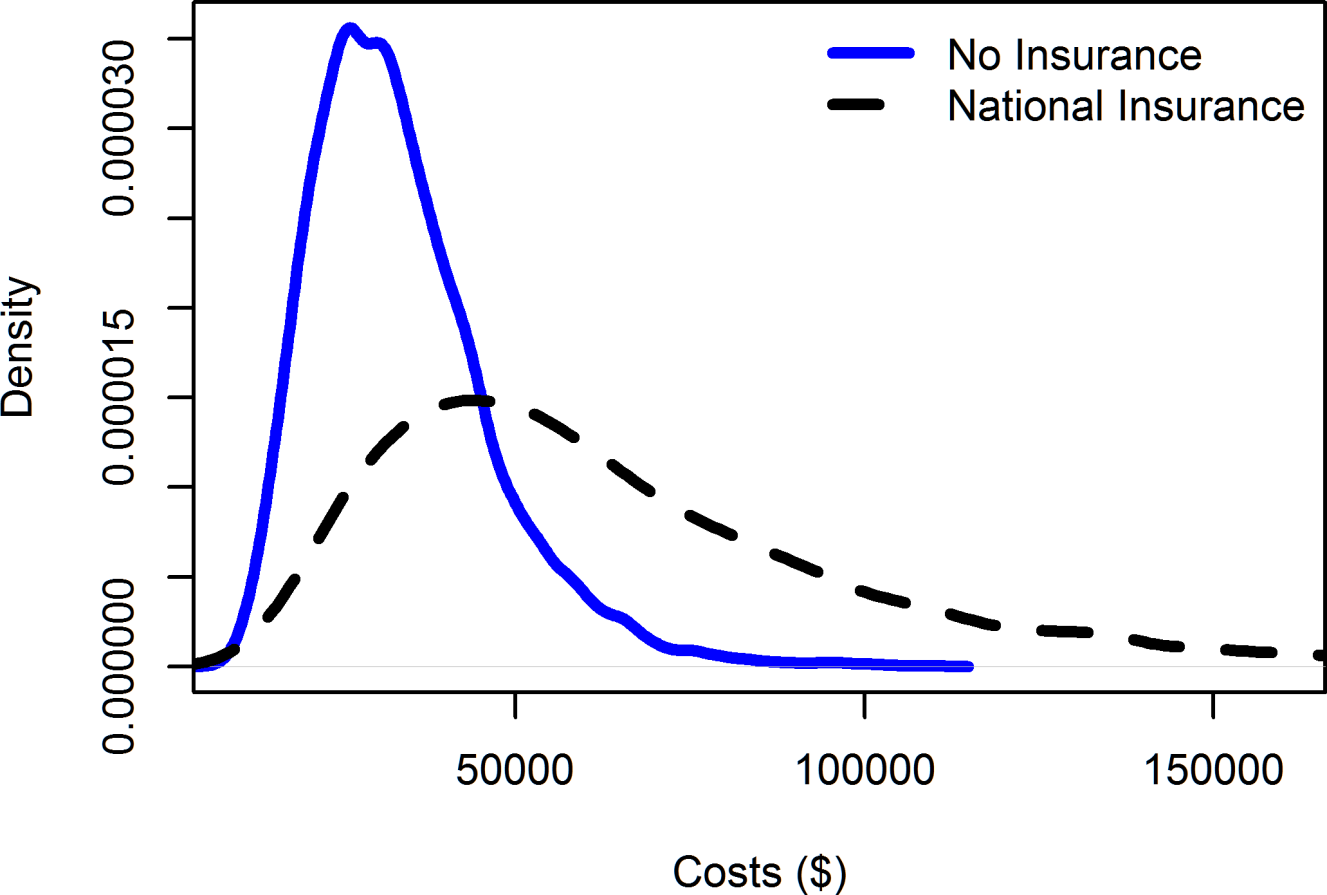
Simulated distributions of the costs of healthcare by health insurance status.

Two studies [9, 11] had zero costs for the control group, as the authors did not include the cost of the ‘status quo’ scenario. Since we have only four selected studies, the zero cost for control groups substantially decreases the mean cost and hence may create biases in the result synthesis. Thus, we replaced the control group costs in these studies with the average cost of control groups in the remaining studies. To keep the cost difference unchanged, we also added the mean cost to the cost of their respective treatment groups. The simulation results show that the average cost for control groups was about $30,000, while the cost for insured groups was $65,000 (Fig 2).

**Fig 3:**
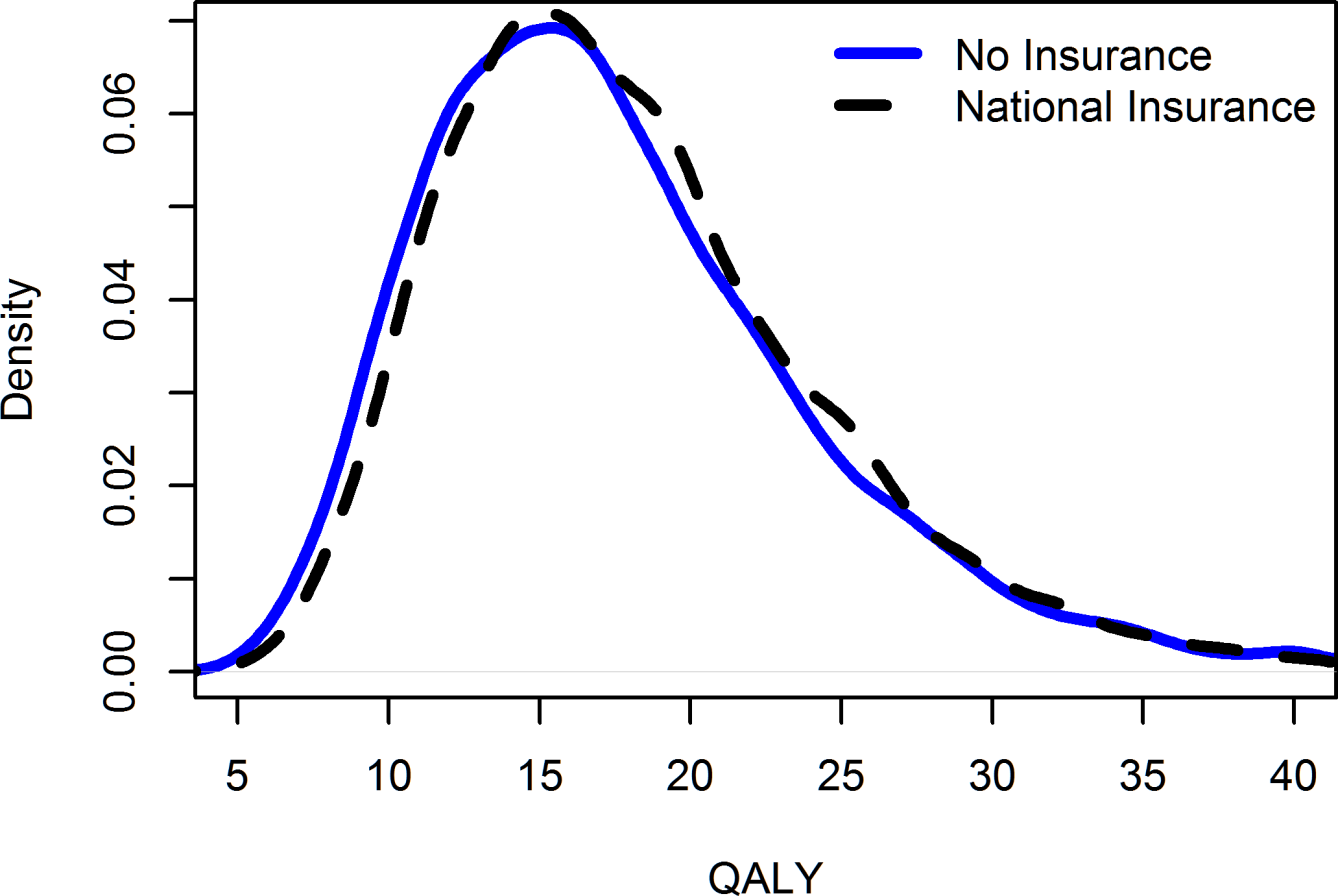
Simulated distributions of the discounted lifetime quality of life by health insurance status. Higher scores indicate a better quality of life.

The introduction of national health insurance programs produced some improvement in the lifetime QALY, except for those at the extreme ends of the distribution (i.e., the very sick or very healthy). Since the data extracted from the selected studies represent the average QALY of the population in these studies, it is unlikely that we have extremely low or extremely high lifetime QALY. On average, people with health insurance had a discounted lifetime QALY, 0.7 higher than those without health insurance (see Fig 3).

**Fig 4:**
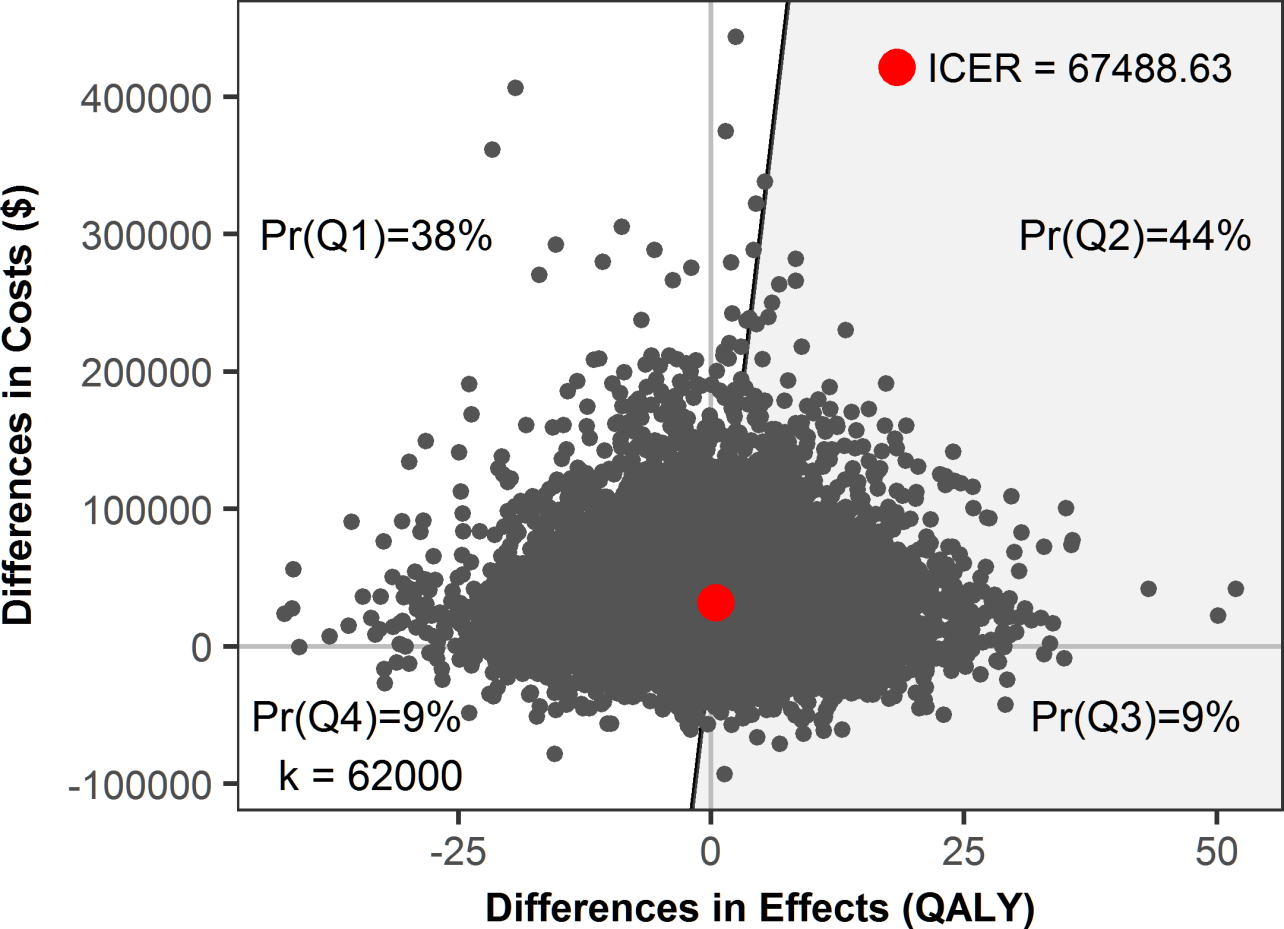
Simulated cost-effectiveness plane. The gray dots are individual simulations and the large red dot is the average. The shaded area below and to the right of the diagonal line is the cost-effective region.

The distributions of costs and QALYs show right-skewness in both the insured and uninsured groups; hence, we mitigated this issue by using a log-normal distribution. On average, the estimated incremental cost-effectiveness ratio of national health insurance is US$51,300. The 95% confidence interval of the ICER, calculated using the Taylor series expansion approach [20], ranges from US$35,800 to US$68,800, which is considerably higher than the most relevant threshold in the UK, at £18,317 (2008 prices) per QALY (about US$28,000 at 2013 prices) [21]. National health insurance increases the discounted lifetime QALY by 0.61 on average, with an additional cost of US$31,294. Using a willingness-to-pay per life-year gained of US$62,000 for the US [18], the evidence synthesis suggests that national insurance programs are cost-effective (Fig 4).

This finding is in line with a recent economic analysis [22], which suggests that providing national health insurance to the population produces a substantial net gain in welfare. The simulation results estimate that 44% of observations lie in the second quadrant of higher costs and higher QALY; 38% lie in the first quadrant of higher costs but lower QALY; 9% lie in the third quadrant of lower costs but higher QALY; and 9% lie in the fourth quadrant of lower costs and lower QALY.

**Fig 5:**
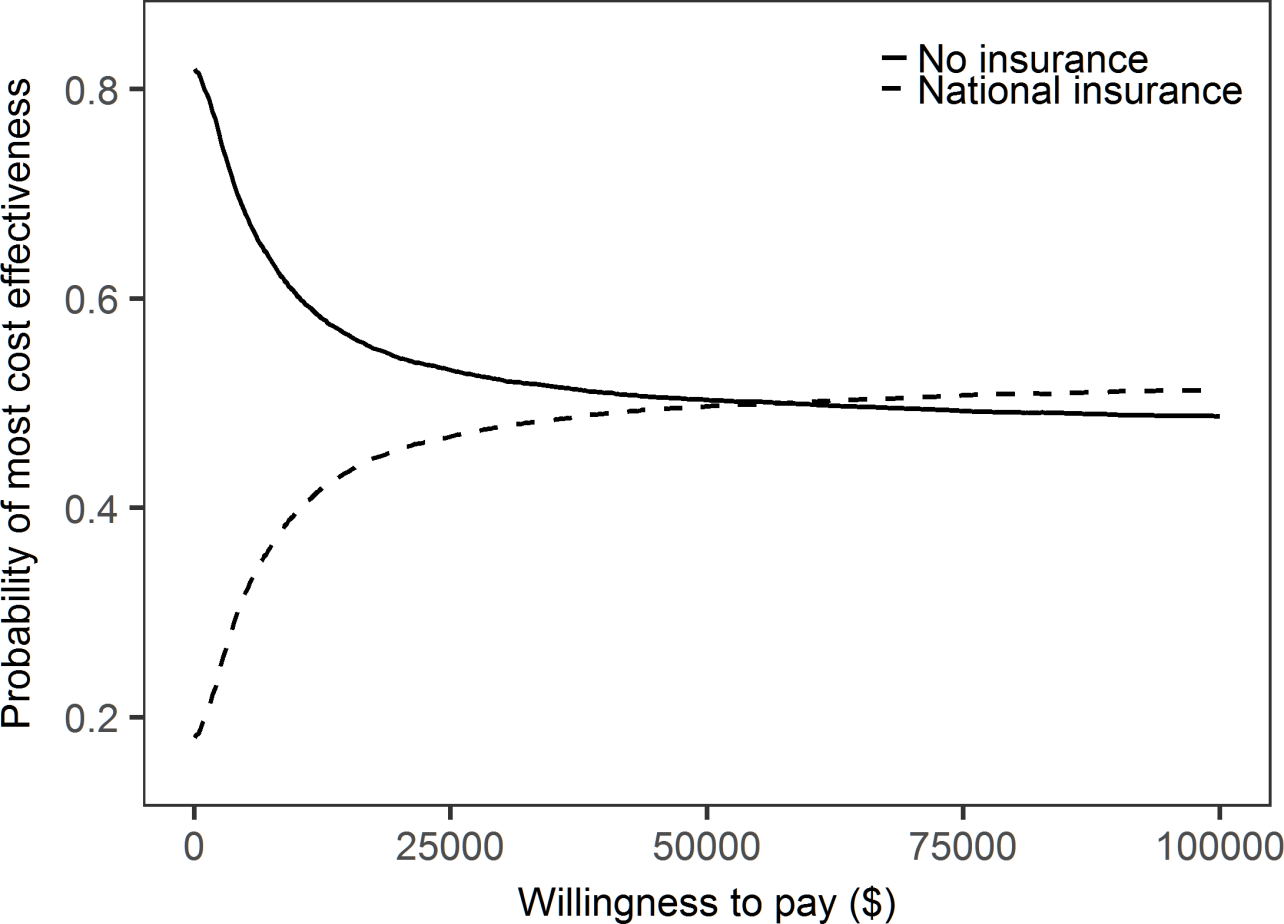
Probability of most cost-effective intervention.

The probability that national health insurance programs will be more cost-effective increases with the willingness-to-pay, while the reverse occurs with no insurance (Fig 5). When the willingness-to-pay reaches $51,300, the provision of national health insurance is more cost-effective than no insurance.

## Discussions

National health insurance programs have been implemented in most developed countries, but there is little evidence of their cost-effectiveness in the literature. Ironically, all studies included in this systematic review are found in the US, a country that only introduced a national health insurance coverage via “Obamacare” in 2010 [23]. Studies of long-standing schemes in Europe could not have been included in this review because there would be no control group in countries that already have universal coverage. The included studies examined the cost-effectiveness of expanding the Medicare or Medicaid programs, which cover vulnerable segments of the population. All studies found that the provision of health insurance is cost-effective, with the ICER range from $24,000 to $64,000. The evidence synthesis confirms this finding with an average change to costs of $51,300 per extra QALY gained.

While not a particularly strong result for cost-effectiveness, it falls below the threshold values of $62,000 per QALY found by Shiroiwa et al. [24] who used a choice experiment to elicit a maximum willingness-to-pay value from 1,000 US citizens. A national insurance scheme may also address equity goals of governments, by providing health care services for groups who were previously uninsured or underinsured. Lower socio-economic groups with poorer health outcomes and more complex needs for services may now be accessing more healthcare. Yet this comes at a relatively higher cost than those covered under the previous health funding arrangements. The data in Fig 2 lend some support to this, with the distribution of costs ‘No insurance’ having a lower mean value, and fewer individuals contributing to the long tail of high-cost outcomes.

Other social objectives are likely to be met by the adoption of national health insurance, such as improved participation in the workforce, leading to productivity gains. Additionally, there may be improved opportunities to regulate health services if they are funded by the public purse. Some of the worst aspects of market failure could be avoided and the misallocation of scarce resources to low-value care or even harmful care might be reduced if third-party payers can exert pressure on providers to be efficient with scarce resources. For example, the introduction of the Affordable Care Act in the United States is associated with 17 percent reduction in the rate of hospital-acquired conditions [25].

While this review focuses on developed countries, emerging economies such as China, Vietnam and India may receive useful insights. Following demographic and epidemiological transitions, universal health insurance is the third global health transition, and countries are encouraged to adopt successful lessons from other regions in their programs [26]. There is a lot of evidence that universal health insurance leads to improvement in population health [27] and economic development [28]. Thus, the result found in this review, that national health insurance is cost-effective, will encourage emerging economies to finance their health system using tax revenue.

## Supporting information

S1 Table Search results. (DOCX)

S2 Table Summary of selected studies. (DOCX)

S3 Table PRISMA checklist. (DOCX)

S1 Fig PRISMA flow diagram. (DOCX)

S1 File Data extraction_results_synthesis.xlsx. (XLSX)

S2 File Result_synthesis2.R. (R)
