# Correction: Cost-effectiveness of national health insurance programs in high-income countries: A systematic review

**DOI:** 10.1371/journal.pone.0191989

**Published:** 2018-01-25

**Authors:** Son Nghiem, Nicholas Graves, Adrian Barnett, Catherine Haden

There is an error in Fig 4. The correct value for ICER is 51301.27. Please see the correct Fig 4 here.

**Fig 4:**
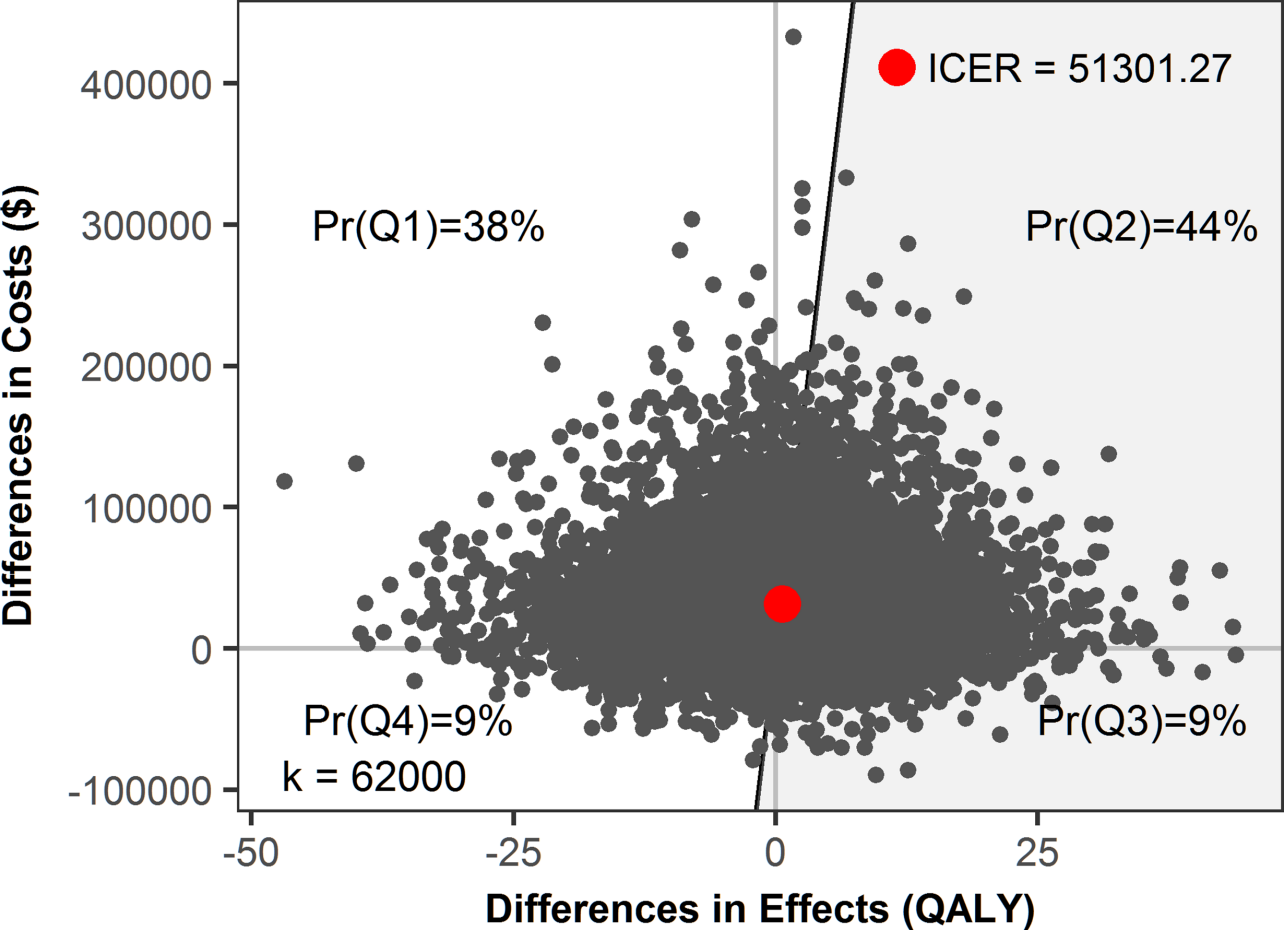
Simulated cost-effectiveness plane. The gray dots are individual simulations and the large red dot is the average. The shaded area below and to the right of the diagonal line is the cost-effective region.
