# Supplementary material for: Cost-effectiveness of national health insurance programs in high-income countries: A systematic review
Source: PLoS One. 2017 Dec 15;12(12):e0189173. doi: 10.1371/journal.pone.0189173 (PMC5731747; doi:10.1371/journal.pone.0189173)
Supplement: S1 Table — (DOCX) [file pone.0189173.s001.docx]

**Table S1. Searches results** (last updated October 2015)

| Search | Results |
| --- | --- |
| PUB MED |  |
| (cost benefit* OR cost effectiveness OR economic*) AND (disability insurance[Title/Abstract] OR national health insurance[Title/Abstract]) | 1705 |
| EMBASE |  |
| cost:ab,ti AND benefit*:ab,ti OR cost:ab,ti AND effectiveness:ab,ti OR economic*:ab,ti AND (disability:ab,ti AND insurance:ab,ti OR national:ab,ti AND health:ab,ti AND insurance:ab,ti) | 2128 |
| Web of Science |  |
| ("cost benefit*" OR "cost effectiveness" OR economic*) AND ("disability insurance" OR "national health" OR "health insurance") NOT (drug* OR obesity OR diabetes OR cancer OR heart OR wound* OR disease OR illness) | 20308 |
| Medline (via Ebscohost) |  |
| ("cost benefit*" OR "cost effectiveness" OR economic*) AND ("disability insurance" OR "national health" OR "health insurance") NOT (drug* OR obesity OR diabetes OR cancer OR heart OR wound* OR disease OR illness) | 16211 |
| Scopus |  |
| ("cost benefit*" OR "cost effectiveness" OR economic*) AND ("disability insurance" OR "national health" OR "health insurance") ABS | 6538 |
| CINAHL via Ebscohost |  |
| ("cost benefit*" OR "cost effectiveness" OR economic*) AND ("disability insurance" OR "national health" OR "health insurance") NOT (drug* OR obesity OR diabetes OR cancer OR heart OR wound* OR disease OR illness) | 7860 |
| ECONLIT via Proquest |  |
| ab(("cost benefit*" OR "cost effectiveness" OR economic*) ) AND ab(("disability insurance" OR "national health" OR "health insurance")) | 811 |
| RePEc |  |
| ("cost benefit*" OR "cost effectiveness" OR economic*) AND ("disability insurance" OR "national health" OR "health insurance") in working papers, articles. | 1277 |
| Cochrane database |  |
| (("cost benefit*" OR "cost effectiveness" OR economic*) AND ("disability insurance" OR "national health" OR "health insurance")) | 129 |
| DARE, NHS EDD and HTA |  |
| (("cost benefit*" OR "cost effectiveness" OR economic*) AND ("disability insurance" OR "national health" OR "health insurance")) | 1399 |
| Campbell Collaboration |  |
| (("cost benefit*" OR "cost effectiveness" OR economic*) AND ("disability insurance" OR "national health" OR "health insurance")) | 27 |
| Health Economics Evaluation Database (HEED) |  |
| (("cost benefit*" OR "cost effectiveness" OR economic*) AND ("disability insurance" OR "national health" OR "health insurance")) | 29 |
