# Supplementary material for: Cost-effectiveness of national health insurance programs in high-income countries: A systematic review
Source: PLoS One. 2017 Dec 15;12(12):e0189173. doi: 10.1371/journal.pone.0189173 (PMC5731747; doi:10.1371/journal.pone.0189173)
Supplement: S2 Table — (DOCX) [file pone.0189173.s002.docx]

**Table S2. Summary of selected studies**

| Authors | Year | Country | Type of study | Estimation | Outcome |
| --- | --- | --- | --- | --- | --- |
| von Wachter et al. | 2008 | United States | Econometric analysis | Double difference | Employment |
| Cutler et al. | 2006 | United States | Econometric analysis | Standard regression | Cost of life year |
| Weathers& Stegman | 2012 | United States | Econometric analysis | Before-after | SF36 (MCS) |
| Bailey and weather | 2014 | United States | Econometric analysis | With-without | Employment |
| Marie & Vall Castello | 2011 | Netherlands | Econometric analysis | Others | Elasticity of labor participation |
| Keng & Sheu | 2013 | Taiwan | Econometric analysis | Double difference | Death hazard |
| Campolieti | 2004 | Canada | Econometric analysis | Double difference | Labor supply |
| Bharmal & Thomas | 2005 | United States | Econometric analysis | With-without | SF12 (PCS) |
| Maestas et al. | 2014 | United States | Econometric analysis | Before-after | Disability application |
| Baicker et al. | 2014 | United States | Cost-effectiveness analysis | Others | Earnings |
| Sommers et al. | 2012 | United States | Others | Double difference | Mortality (deaths/100 k pop) |
| Engel | 1994 | Germany | Others | Others | Drug cost |
| Michalopoulos et al. | 2012 | United States | Others | Randomized | Unmet medical need |
| Sheu et al. | 2014 | Taiwan | Others | Double difference | Consumption (spillover effects) |
| Muennig et al. | 2015a | United States | Cost-effectiveness analysis | With-without | Cost effectiveness ratio |
| Muennig et al. | 2015b | United States | Cost-effectiveness analysis | Others | Cost effectiveness ratio |
| Franks et al. | 2005 | United States | Cost-effectiveness analysis | Others | Cost effectiveness ratio |
| Muennig et al. | 2005 | United States | Cost-effectiveness analysis | Others | ICER |
