# Supplementary material for: Cost-effectiveness of national health insurance programs in high-income countries: A systematic review
Source: PLoS One. 2017 Dec 15;12(12):e0189173. doi: 10.1371/journal.pone.0189173 (PMC5731747; doi:10.1371/journal.pone.0189173)
Supplement: S1 Fig — (DOCX) [file pone.0189173.s004.docx]

**Figure S1. PRISMA flow diagram**

Search record through reference databases: 58,422

Identification

Excluded 14 full-text articles because:

- *Conduct econometric analyses rather than cost-effectiveness*
- *Use other outcomes than QALY*

Screening

Eligibility

Full-text articles eligible for detailed examination: 18

Included

4 US studies included in final analyses

- *Two studies of Medicare*
- *One study of Medicaid*
- *One study of health reforms*

22,656 records were excluded because:

- *Did not have ‘health insurance’ or ‘disability’ equivalent in the title*
- *Did not have ‘cost effectiveness’ or ‘cost utility’ in the title*
- *Focus on some diseases/conditions*
- *Commentary rather than analysis*

Removal of duplicates: 35,748

Unique records remain for screening: 22,674
